# NK cells in renal cell carcinoma and its implications for CAR-NK therapy

**DOI:** 10.3389/fcell.2025.1532491

**Published:** 2025-02-20

**Authors:** Xinwei Li, Yuanpeng Zhang, Yuzhong Ye, Wen Xiao, Lei Liu, Xiaoping Zhang

**Affiliations:** ^1^ Department of Urology, Union Hospital, Tongji Medical College, Huazhong University of Science and Technology, Wuhan, China; ^2^ Institute of Urology, Union Hospital, Tongji Medical College, Huazhong University of Science and Technology, Wuhan, China; ^3^ Department of Urology, Fujian Medical University Union Hospital, Fuzhou, China; ^4^ Shenzhen Huazhong University of Science and Technology Research Institute, Shenzhen, China

**Keywords:** renal cell carcinoma, NK cell, immune response, CAR-NK, immunotherapy

## Abstract

Renal cell carcinoma (RCC) is a malignancy that makes up 3% of adult cancers and 20%–30% of patients were diagnosed with metastatic RCC in the beginning, while the median overall survival (OS) of metastatic RCC systemic therapy ranges from 16 months to 50 months. Immunotherapy, a novel therapy that relies on the specific binding of immune cells and tumor cells, may be a potential therapy for advanced renal cell carcinoma. While chimeric antigen receptor NK-cell (CAR-NK) therapy has been investigated in a variety of solid tumors, specific research on its application to RCC has also been reported by several teams. In this review, we introduced the cytotoxicity mechanisms of NK cells, summarized the connections between RCC and NK cells, and posted new insights into renal cell carcinoma CAR-NK therapy. To date, most researches focusing on renal cell carcinoma and NK cells only claimed the mechanisms of NK cell cytotoxicity and NK cell immune suppression and even immune escape, yet the molecules involved could also be interesting targets for renal cell carcinoma CAR-NK therapy.

## 1 Introduction

Renal cell carcinoma (RCC) is a malignancy that makes up 90%–95% of the kidney cancers and 3% of adult cancers (Catalano et al., 2023). It has been estimated that about 81,800 new cases and 14,890 deaths would be found in the United States in 2023 (Siegel et al., 2023). According to pathological examinations, RCC could be classified as clear cell renal cell carcinoma (ccRCC), papillary RCC, chromophobe RCC, translocation-associated RCC, medullar RCC, and collecting duct RCC, while ccRCC is the most common type and accounts for 75%–85% of RCC cases (Tykodi et al., 2022; Wu et al., 2023). Though nearly 80% of patients were diagnosed with localized RCC and radical surgery treatment was performed after diagnosis, 20% of them would progress in the follow-up, whereas around 20%–30% of patients were diagnosed with metastatic RCC in the beginning (Wiechno et al., 2018). In the past 20 years, RCC systemic therapy mainly relies on targeted therapies (TTs) and immune checkpoint inhibitors (ICIs) (Srivastava et al., 2022). The former contains inhibitors of vascular endothelial growth factor (VEGF) signaling such as sunitinib and inhibitors of hypoxia inducible factor (HIF) pathway including belzutifan, while the latter includes programmed death-1 (PD-1), programmed death ligand-1 (PD-L1), and the cytotoxic T-lymphocyte associated protein-4 (CTLA-4) (Cardenas et al., 2022). However, the median overall survival (OS) of metastatic RCC systemic therapy still ranges from 16 months to 50 months (Dudani et al., 2021).

Natural Killer (NK) cells, characterized by killing tumor cells by different means without previous sensitization, have played a key role in the tumor immunity (Terrén et al., 2019). The main mechanisms of NK cell-mediated killing could be categorized as “missing-self” and antibody-dependent cell-mediated cytotoxicity (ADCC) (Myers and Miller, 2021). Currently, immunotherapy focusing on increasing NK cell antitumor immunity has brought new insights into cancer therapy (Deuse et al., 2021). The chimeric antigen receptor NK-cell (CAR-NK) therapy has already been studied in solid tumors such as head and neck squamous cell carcinoma (HNSCC), non-small cell lung cancer (NSCLC), triple-negative breast cancer (TNBC), glioblastoma, and hepatocellular carcinoma (HCC) (Ciulean et al., 2023; Fang et al., 2023b; Raftery et al., 2023; Strecker et al., 2022; Tseng et al., 2020). Till now, there were only a few reviews that summarized the promising utilizations of immunotherapy in RCC, while no review focused on the relationship between RCC and NK cell-based immunotherapies, especially CAR-NK therapy. In this review, we introduced the cytotoxicity mechanisms of NK cells, summarized the connections between RCC and NK cells, and posted new insights into RCC CAR-NK therapy, which may enlighten the present researches in RCC immunotherapy.

## 2 Basic function of NK cells

NK cells are one of the important components of the innate immune system and the first line defense against various diseases including tumors (Becker et al., 2016).

**FIGURE 1:**
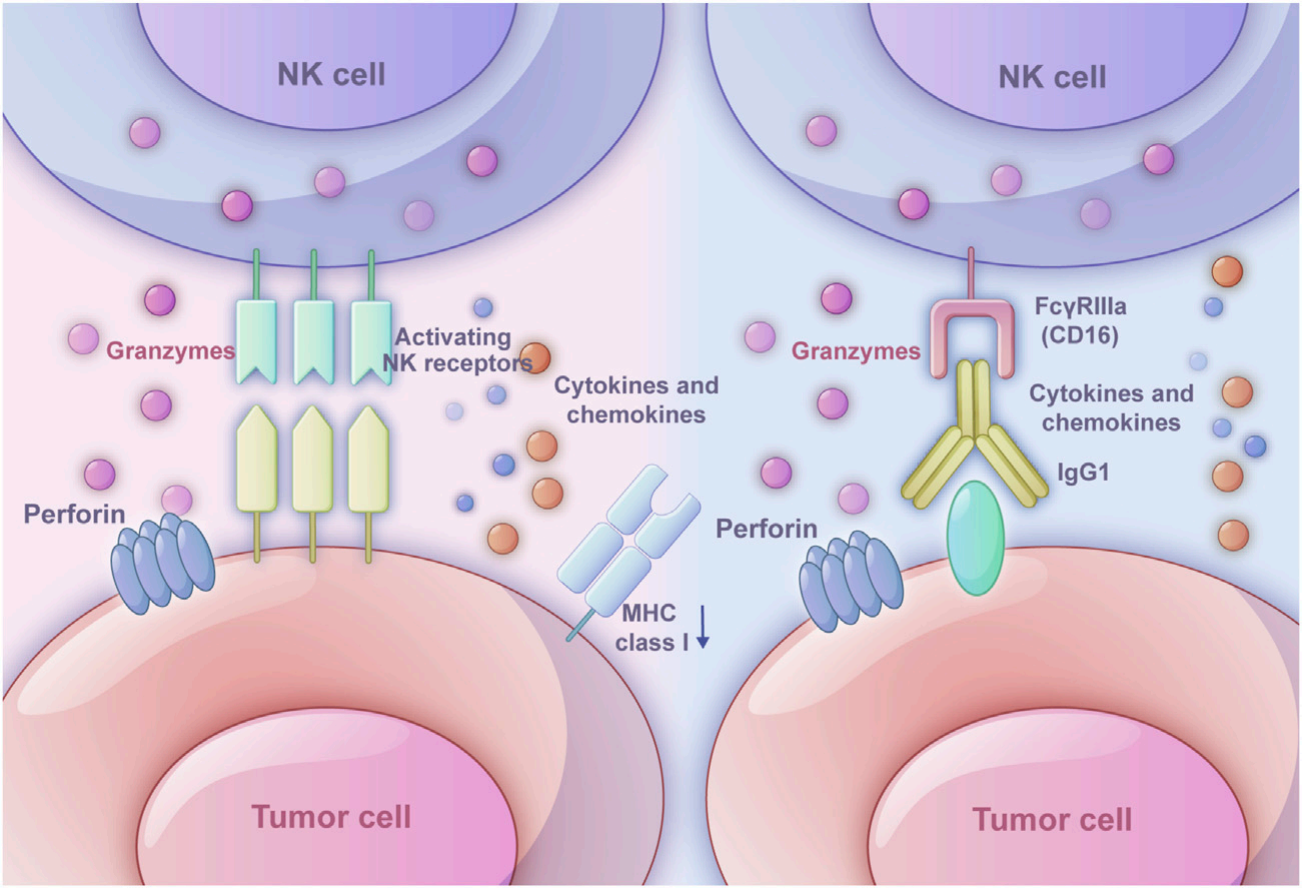
Two ways in which NK cells triggers immune response. The left one is the ‘missing-self’ based on inhibitory receptors and activating receptors. When MHC class I expression on target cells is reduced or absent, the inhibitory signals weaken, and activating signals dominate, leading NK cells to exhibit cytotoxic effects. The right one is antibody-dependent cell-mediated cytotoxicity (ADCC). The Fab segment of an antibody specifically binds to molecules on the tumor cell surface, while the CD16 receptor on NK cells binds to the Fc segment of IgG1 antibodies. Once the target cell is recognized, NK cells activate exocytosis of cytotoxic granules or death receptor-mediated cytotoxicity. Perforin forms pores in the target cell membrane, allowing granzymes to enter and induce apoptosis.

NK cell receptors could be divided into inhibitory receptors and activating receptors. The inhibitory receptors include killer cell immunoglobulin-like receptor, two immunoglobulin domains, and long cytoplasmic tail (KIR-2DL), killer cell immunoglobulin-like receptor, three immunoglobulin domains, and long cytoplasmic tail (KIR-3DL), CD94/NKG2A, and T-cell Immunoreceptor with Ig and ITIM domains (TIGIT). The activating receptors mainly contain killer cell immunoglobulin-like receptor, two immunoglobulin domains, and short cytoplasmic tail (KIR-2DS), killer cell immunoglobulin-like receptor, three immunoglobulin domains, and short cytoplasmic tail (KIR-3DS), natural cytotoxicity receptor (NCR) (NKp46, NKp44, and NKp30), natural killer group 2, member D (NKG2D), SLAM family member 4(2B4), CD226, CD94/NKG2C. Most inhibitory receptors inhibit NK cell function by recognizing MHC class I molecules, which could transmit inhibitory signals and participate in autoimmune tolerance under physiological conditions to avoid killing normal cells. However, when MHC class I molecules on target cells are weakened or absent, or specific ligands directly recognize activating receptors, the inhibitory signal weakens and the activation signal enhances, leading to NK cells exhibiting cytotoxic effects (Chen et al., 2020). Based on these receptors, NK cells are able to recognize and rapidly attack against malignant cells without prior sensitization and impairing normal cells (Vivier et al., 2011) (Figure 1).

ADCC relies on the interaction among NK cells, antibodies, and tumor cells. The Fab segment of the antibody could specifically bind specific molecules on the surface of tumor cells, which modulates the function in an agonistic or antagonistic manner eliciting ADCC effect (Dixon et al., 2021). The binding of the FcγRIIIa (CD16) on NK cells and the Fc segment of IgG1 antibodies relies on the interaction between the hinge region and methylene structural domain (Li and Liu, 2022). After recognizing the target cell, the exocytosis of cytotoxic granules or death receptor-mediated cytotoxicity of NK cells would be activated. As a common cytotoxic granule, perforin would generate pores in the target cell membrane, which allows granzymes to enter the cell and induce the apoptosis through caspase-dependent and caspase-independent pathways (Grudzien and Rapak, 2018). The death receptor-mediated cytotoxicity starts with the formation of death-inducing signaling complex, which is composed of activated death receptors and recruited Fas-associated death domain (FADD) adaptor proteins and initiator procaspases 8–10 and later will initiate a caspase cascade and induce target cell apoptosis (Prager et al., 2019). Besides, owing to the production of the cytokine IFNγ, NK cells could directly kill target cells through cytotoxic mechanisms (O'Brien and Finlay, 2019) (Figure 1).

## 3 NK cells in RCC immune response

In the RCC immune response, NK cells in normal kidney tissue and in the RCC region exhibited heterogeneity and the normal kidney tissue may be a NK cell reservoir of RCC region for immune response (Strizova et al., 2019). The peripheral NK (pNK) cells in the normal region were CD56^+^CD16bright that lacked full cytotoxic ability, while tumor-infiltrated NK (TiNK) cells were CD56^+^CD16dim-neg cells, a phenotype of decidua NK (dNK) cells, that exhibited possibility of conversion from pNK cells and increasing angiogenic and inflammatory genes with enrichment of genes in the HIF-1α pathway (Guan et al., 2020). Besides, NK cells in RCC region could also elevate their expression of lymphocyte activation gene 3 (LAG-3), PD-1, and HLA-DR (Lee et al., 2022). From another aspect, the NK cells in the ccRCC and normal kidney tissues could be classified into three subpopulations as NK(GZMH), NK(EGR1), and NK(CAPG). Among these three subtypes, NK(EGR1) and NK(CAPG) were mainly in RCC region closely related to RCC metastasis (Liang et al., 2022).

The cytotoxicity of NK cells in RCC tissues could also be enhanced by various molecules via signaling pathways and ligand-receptor recognition. A study found that von Hippel-Lindau (VHL)-mutated RCC tumors exhibited infiltration by NKp46+ cells and showed higher expression of the NKp30 and NKp46 receptors compared to VHL-wild type RCC tumors, which means that the increased expression of activating NK receptor ligands in VHL-mutated RCC tumors, alongside potential downregulation or loss of MHC class I molecules, indicates a promising approach to enhancing NK cell-mediated antitumor immunity (Trotta et al., 2018). Several novel HLA-G-regulatory miRs, miR-548q, miR-628-5p, miR-148A, and miR-133A could cause downregulation of HLA-G mRNA and protein, resulting in an enhanced NK cell-mediated HLA-G-dependent cytotoxicity (Jasinski-Bergner et al., 2016; Jasinski-Bergner et al., 2015). Besides, circZKSCAN1 knockdown potentiated NK cell cytotoxicity against RCC and repressed tumor growth (Li et al., 2022). CD25bright NK cells isolated from IL-15 primed NK cells presented an increasing proliferative and metabolic activity via mTOR pathway with an increasing ability to Treg cells containing RCC tumor spheroids compared with CD25dim NK cells (Chen et al., 2023). Axitinib, a kind of tyrosine kinase inhibitor, could increase the expression of NKG2D on NK cells via DNA damage response (DDR) induction, which promoted NK cell recognition and degranulation in a reactive oxygen species (ROS)-dependent manner in RCC immune response (Morelli et al., 2015).

On the contrary, several molecules could also lead to RCC immune suppression and even immune escape via signaling pathways and ligand-receptor recognition, and most of these molecules have a close connection with inhibitory receptors and MHC class I molecules. For instance, the VHL gene in RCC could downregulate hypoxia-inducible factor (HIF)-1α and subsequently decrease vascular endothelial growth factor (VEGF) production, while VHL mutation in RCC could also enhance HLA-I expression and IFNα resistance (Perier et al., 2011). Another membrane type matrix metalloproteinase 2 (MMP2) could mediate the MHC class I-chain related protein A (MICA) shedding, which led to the release of soluble MICA (sMICA) facilitating the RCC immune escape (Yang et al., 2014). In addition, NK-derived IFN-γ would lead to the upregulation of RCC MHC class I, thus causing resistance to NK cytotoxicity (Zhuang et al., 2019). Interestingly, another study found that the MICA was overexpressed RCC, and there was also correlation between the NKG2D-MICA axis and the decreasing overall survival of ccRCC patients. MICA was the only NKG2DL that over-expressed in ccRCC cells. The NKG2D was upregulated on tumor-infiltrating NK cells (TINKs) but downregulated on peripheral blood NK cells (PBNKs). Moreover, the TINKs impaired their degranulation that negatively associated with NKG2D expression, diminished IFN-γ production, upregulation of T cell immunoglobulin mucin domain-containing protein 3 (TIM-3), and an impaired glucose intake upon stimulation with cytokines, indicating that they were dysfunctional and displayed features of exhaustion and an altered metabolic fitness (Secchiari et al., 2022). Additionally, through NKG2D directly recognizing RCC cells, PD-L1 expression was induced on NK cells and was further upregulated by monocyte-derived IL-18, thus suppressing the immune response (Sierra et al., 2021). Another interesting finding claimed that KIR2DL4 was also highly expressed in RCC cells, which promotes RCC progression through phosphatidylinositol-3-kinase (PI3K)/protein kinase B (AKT) activation (Ding et al., 2022). Similarly, HERV-H LTR-associating 2 (HHLA2), a molecule that is frequently expressed in various tumors including ccRCC, could mediate immune evasion through binding the inhibitory receptor killer cell immunoglobulin-like receptor, three immunoglobulin domains, and long cytoplasmic tail 3 (KIR3DL3) on the NK cells (Bhatt et al., 2021). Sialic acid-binding Ig-like lectin-7 (Siglec-7) is an inhibitory receptor expressed on NK cells and has a preference for internally branched α2,6-linked disialic gangliosides such as DSGb5 on RCC. A study has found that DSGb5 in RCC could downregulate NK cell cytotoxicity in a Siglec-7-dependent manner and for RCC proliferation and metastases (Kawasaki et al., 2010). DNAX accessory molecule-1 (DNAM-1) is essential to NK cell-mediated cytotoxicity. However, in comparison with the peripheral blood, RCC-infiltrating NK cells reduced their DNAM-1 expression because the RCC cells could downregulate on DNAM-1 NK cells via interaction with the poliovirus receptor (PVR) and may cause immune escape. This has also been proved by the results that intratumoral DNAM-1+ NK cells were negatively correlated with PVR expression levels in RCC tumors (Tong et al., 2023). However, there are still some molecules that cause RCC immune suppression and immune escape through other pathways and have an influence on the NK cell infiltration and cytotoxicity. Ren et al. have claimed that kinesin family member 20A (KIF20A) had a negative correlation with NK cells and its upregulation could promote tumor proliferation and invasion (Ren et al., 2020). The Kruppel-Like Factor 16 (KLF16) could regulate circFOXO3 expression, while circFOXO3 would further regulate NK cell cytotoxicity towards RCC cells by directly sponging miR-29a-3p and miR-122-5p. The overexpressed miR-29a-3p or miR-122-5p may decrease the NK cell toxicity against RCC cells (Yang et al., 2022). In addition, compared with NK cells from tumor margin tissue and non-tumor tissue, the tumor-infiltrating NK cells have poorer cytotoxic capacity and potential to produce cytokines. This was because the RCC-derived exosome could mediate NK cells to a deficient status through TGF-β/SMAD signaling pathway (Xia et al., 2017). DNA polymerase delta 1 catalytic subunit (POLD1), a key molecule in genomic copy and DNA damage repair process, is associated with NK cell infiltration. The POLD1 expression is positively associated with CD56bright NK cell infiltration and negatively related to CD56dim NK cell infiltration. Results have shown that the higher POLD1 expression in ccRCC may imply a higher proportion of CD56bright NK cells, which also meant a stronger immunosuppressive environment in tumor (Tian et al., 2023). Besides, NK cells from ccRCC exhibited high levels of signaling attenuator diacylglycerol kinase (DGK)-α and blunted mitogen-activated protein kinase pathway activation compared to NK cells from normal kidney and peripheral blood, which may display conjoint phenotypic alterations and dysfunction induced by ccRCC tumor. This may possibly explain the immune escape of ccRCC and the survival benefit of patients with high NK cell infiltration (Prinz et al., 2014). What’s more, the solute carrier family 7 member 11(SLC7A11) molecule in RCC could lead to a decline in the immune abundance of NK cells (Xu et al., 2021) (Table 1).

**TABLE 1:** Molecular mechanisms of RCC immune escape or suppression mediated by NK cells.

Molecules	Mechanisms	References
VHL	Downregulates HIF-1α, reducing VEGF production Enhances HLA-I expression and IFNα resistance	Perier et al. (2011)
MMP2	Mediates MICA shedding, leading to the release of soluble MICA (sMICA) and facilitating immune escape	Yang et al. (2014)
IFN-γ	Upregulates MHC class I on RCC cells, causing resistance to NK cytotoxicity	Zhuang et al. (2019)
NKG2D	Induces PD-L1 expression on NK cells, suppressing immune response	Sierra et al. (2021)
HHLA2	Mediates immune evasion by binding to the inhibitory receptor KIR3DL3 on NK cells	Bhatt et al. (2021)
Siglec-7	Downregulates NK cell cytotoxicity via DSGb5 in an Siglec-7-dependent manner, promoting RCC proliferation and metastasis	Kawasaki et al. (2010)
DNAM-1	RCC cells downregulate DNAM-1 expression on NK cells through interaction with PVR, potentially causing immune escape	Tong et al. (2023)
KLF16/circFOXO3	Regulates NK cell cytotoxicity towards RCC cells by sponging miR-29a-3p and miR-122-5p	Yang et al. (2022)
RCC-derived Exosomes	Mediate NK cells to a deficient status through the TGF-β/SMAD signaling pathway	Xia et al. (2017)
POLD1	Positively associated with CD56bright NK cell infiltration and negatively related to CD56dim NK cell infiltration, implying a stronger immunosuppressive environment in tumors	Tian et al. (2023)
DGK-α	High levels in NK cells are associated with blunted MAPK pathway activation, indicating phenotypic alterations and dysfunction induced by ccRCC.	Prinz et al. (2014)
SLC7A11	Leads to a decline in the immune abundance of NK cells	Xu et al. (2021)

## 4 Association of NK cell markers with RCC prognosis

Moreover, some genes associated to NK cells were also prognostic factors of RCC. The NK cells are a key factor affecting ccRCC progression and immune surveillance and nine genes (BID, CCL7, CSF2, IL23A, KNSTRN, RHBDD3, PIK3R3, RNF19B and VAV3) related to NK cells were identified as prognostic factors (Shi et al., 2023). Another study has analyzed several NK cell marker genes and found out 7 genes (CLEC2B, PLAC8, CD7, SH3BGRL3, CALM1, KLRF1, and JAK1) could predict the prognosis of RCC patients within high Fuhrman grade (G3-G4) and American Joint Committee on Cancer (AJCC) stage (III-IV). This high-risk group had a higher tumor mutation burden (TMB), greater infiltration of immunocytes, and higher expression of genes negatively regulating anti-tumor immunity (Wang et al., 2023). These markers help identify patients who are more likely to respond positively to NK cell therapy, thereby facilitating personalized medicine approaches (Liu et al., 2024). By predicting therapeutic efficacy, they can guide the selection of the most effective treatment regimens, such as determining whether to combine NK cell therapy with other immunotherapies to overcome potential drug resistance, which potentially increasing the success rate in treating RCC patients.

## 5 NK cell-based immunotherapy and promising future of CAR-NK

Currently, three experiments have utilized NK cells targeting RCC cells via ligand-receptor recognition and regulating the tumor microenvironment (TME). Tetramethypyrazine (TMP) has been regonized as an anticancer compound against cancers. TMP could activate NK cells through upregulating NKG2D ligands (NKG2DLs) MHC class I chain-related molecules A and B (MICA/B) expression, which could further suppress RCC epithelial-mesenchymal transition (EMT) progression (Luan et al., 2016). Human anti-CAIX mAbs that inhibit carbonic anhydrase (CA) enzymatic activity could kill RCC through NK cell-mediated ADCC, and the killing activity was positively correlated with the level of CAIX expression in RCC (Chang et al., 2015). With the stimulation of glucocorticoid-induced TNFR related protein (GITR), sunitinib would reprogram tumor-associated macrophages (TAMs) towards M1 polarization, which caused NK cell antitumor response via inhibiting STAT3 activity. This finding may remodel RCC microenvironment to trigger regressions of established metastatic cancer (Yu et al., 2016).

**FIGURE 2:**
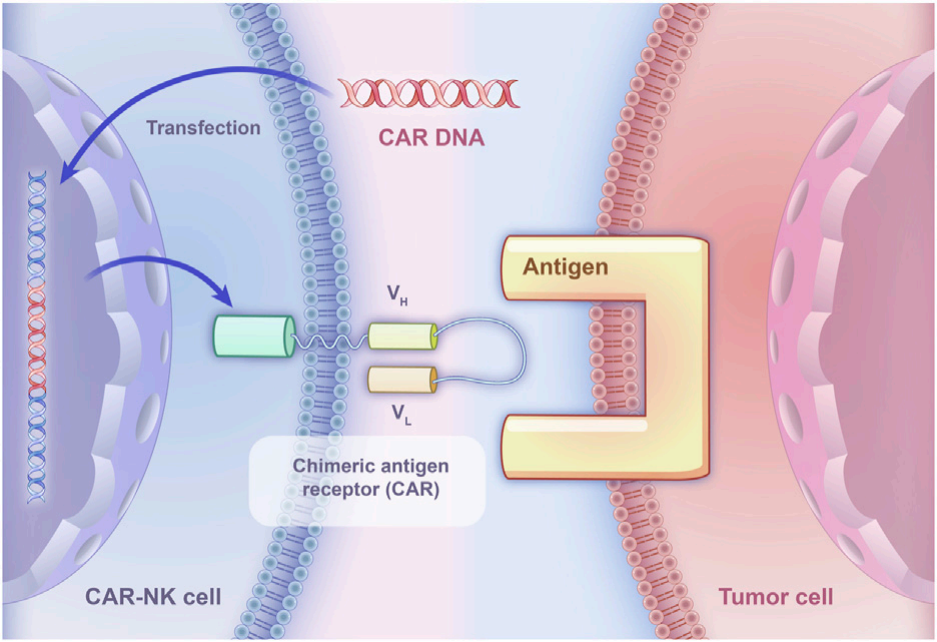
The basic principle of CAR-NK specifically binding to tumor cells. CAR DNA is transfected into NK cells, leading to the expression of chimeric antigen receptors on their surface. The chimeric antigen receptors consist of an extracellular domain derived from a monoclonal antibody that specifically recognizes a tumor-associated antigen, and an intracellular signaling domain that activates the NK cell upon binding to the antigen.

However, due to the RCC immune escape mechanisms, the effectiveness of relying solely on the recognition capabilities of NK cells to kill tumor cells becomes limited. To directly target tumor cells, CAR-NK therapy may be an effective problem solver and CAR-NK has already been applied to targeting some molecules in five RCC experiments. CAR-NK therapy utilizes genetic engineering technology to specifically express CAR on NK cells to bind them to tumor cells, which could kill tumor cells through immune response, thereby achieving the goal of treating malignant tumors (Figure 2). Besides, compared with traditional NK cells equipped with inhibitory receptors for MHC class I molecules, CAR-NK cells are designed to bypass this inhibition, allowing them to effectively target MHC-I+ tumor cells and prevent immunosuppression caused by MHC class I upregulation (Zhuang and Long, 2022).

Renal cell carcinoma exhibited a comparable NK ligand expression profile. The adapter chimeric antigen receptor (AdCAR) NK-92 cells, rather than the parental NK-92 cells, could successfully lyse renal cell carcinoma cells in less than 4 h within the presence of specific biotinylated antibodies (bAb). In standard *in vitro* cytotoxicity assays, AdCAR NK-92 cells effectively eliminated tumor cells from newly established cell lines derived from renal cell carcinoma bone metastasis within 2 hours (Grote et al., 2021). The therapeutic effect of CAR-NK on solid tumors may be limited, but the combination of CAR-NK cells and chemotherapy drugs may be a promising strategy in treating solid tumors. An epidermal growth factor- (EGFR-) specific third-generation CAR was developed by Zhang et al., and the specific killing ability of CAR-modified NK-92 cells (CAR-NK-92) against renal cell carcinoma cell lines has been confirmed *in vitro*. The synergistic effect of cabozantinib and EGFR-specific CAR-NK-92 cells was studied *in vitro* and *in vivo*, and the CAR-NK-92 cells could lyse RCC cells in an EGFR-specific manner. Moreover, cabozantinib can increase EGFR in RCC cells and reduce PD-L1 membrane surface expression, enhancing the killing ability of CAR-NK-92 cells against renal cell carcinoma (Zhang et al., 2017). The same research team also transduced carbonic anhydrase IX (CAIX)-specific third-generation CAR into NK92 cells using lentiviral vectors. Results have shown that CAIX-specific CAR-NK-92 cells could specifically recognize CAIX-positive RCC cells cultured *in vitro* and release cytokines including IFN-γ, perforin, and granzyme B that exhibit specific cytotoxicity. Similarly, after treating RCC cells with bortezomib *in vitro*, the cytotoxicity of CAR-NK-92 cells was enhanced (Zhang et al., 2018). Another study has focused on evaluating the cytotoxicity of a third-generation NK cell CAR against CD24 in RCC. Treating RCC cells with NK-CD24-CAR cells could lead to decreased cell viability and induction of apoptosis, especially in CD24+ tumor cells (Söhngen et al., 2023). Besides, another team has established human NK-92 cells that could recruit them to the tumor associated ErbB2 (HER2) antigen. They have generated a stable clone cell line expressing a humanized CAR based on ErbB2-specific antibody FRP5 harboring CD28 and CD3ζ signaling domains (CAR 5.28.z). These NK-92/5.28. z cells could effectively lyse tumor cells expressing ErbB2 *in vitro* and the retention of specific recognition and anti-tumor activity towards tumor cells *in vivo* resulted in a reduction in lung metastasis in RCC models. Besides, γ-irradiation, a potential safety measure for clinical application, could prevent the replication of NK cells while maintaining anti-tumor activity (Schönfeld et al., 2015) (Table 2).

**TABLE 2:** Summary of present CAR-NK therapy under research.

Targets	CAR-NK cell types	Combination therapies	References
Linker-label epitope	AdCAR NK-92 cells	—	Grote et al. (2021)
EGFR	Third-generation CAR-NK-92 cells	Cabozantinib	Zhang et al. (2017)
CAIX	Third-generation CAR-NK-92 cells	Bortezomib	Zhang et al. (2018)
CD24	Third-generation CAR-NK cells	—	Söhngen et al. (2023)
HER2	NK-92/5.28.z cells	—	Schönfeld et al. (2015)

Ideally, the target antigen should exhibit high expression levels in tumor cells and minimal to no expression in normal cells. Furthermore, it should be localized on the cell membrane to ensure effective recognition and targeting by CAR-NK cells (Li et al., 2024). For ccRCC, CAIX stands out as an ideal target antigen for CAR-NK therapy. CAIX is overexpressed on RCC tumor tissue, while it exhibits no expression on normal cells, thereby minimizing the risk of off-tumor toxicity. Its localization on the cell membrane makes it readily accessible and recognizable by CAR-NK cells, facilitating targeted lysis of tumor cells (Fang et al., 2023a).

However, though existing some lab experiments, at present, only eight clinical trials about NK cell-based immunotherapies for renal cell carcinoma are found on https://clinicaltrials.gov. Five of them rely on the combination of NK cell-based immunotherapy and other immunomodulators. NCT00328861 focused on the safety and efficacy of NK cells and IL-2 in treating metastatic renal cell carcinoma. NCT03319459, NCT05069935, and NCT04551885 were three studies on the therapeutic effect of FATE-NK100, FT538, and FT-516 combined with monoclonal antibodies on solid tumors. NCT06318871 studies the feasibility of CIML NK cell therapy combined with N-803 in the treatment of advanced clear cell renal cell carcinoma. This is the first time that a specific combination of CIML NK cells and NIZ803 (IL-15 superantagonist) will be given to human. Two clinical trials, NCT04106167 and NCT03841110, were immunotherapies based solely on FT500 NK cells. There is only one clinical trial for CAR-NK therapy. NCT05703854 bases on CAR-NK therapy to determine the safety, tolerability, and optimal cell dose of chimeric antigen receptor (CAR).70/interleukin (IL)15-transduced cord blood (CB)-derived NK cells in patients with advanced renal cell carcinoma. This is a phase I/II clinical trial and it is still recruiting, so clinical data and results need to be awaited (https://clinicaltrials.gov).

So far, there has only been one clinical trial involving CAR-NK to treat RCC on the clinicaltrials.gov., which may due to various limitations. CAR-based immunotherapy faces multiple challenges in treating solid tumors, including the identification of truly specific tumor antigens, overcoming antigen escape mechanisms, improving CAR cell trafficking and infiltration to the tumor site, and ensuring their persistence and functionality within the suppressive TME. Additionally, CAR-NK cells must address transduction efficiency while preserving their natural cytotoxicity and functional attributes (Maalej et al., 2023; Zhou et al., 2023). Another article has addressed several drawbacks that may hinder the successful application of CAR-NK cells in treating solid tumors. These include resistance to viral transduction and a short lifespan with poor persistence. NK cells exhibit lower and more variable transduction efficiency compared to T cells, which can impact the effectiveness and statistical power of clinical trials. Additionally, γ-retroviruses used for transduction pose a higher risk of insertional mutagenesis. The short half-life and limited persistence of NK cells *in vivo* may necessitate multiple infusions, potentially affecting the cost-effectiveness and safety of the therapy (Peng et al., 2024). Therefore, the vast majority of CAR-NK researches are still mainly focused on the cell lines. However, a significant finding from a clinical trial of B cell tumors is that CAR19/IL-15 CBU-NK cells are not associated with notable cytokine release syndrome (CRS) or neurotoxicity. This contrasts sharply with CAR-T cell therapies, which often come with higher risks of severe CRS and neurotoxicity, suggesting CAR-NK cells may offer a safer therapeutic option (Marin et al., 2024).

## 6 Conclusion

Renal cell carcinoma especially advanced renal cell carcinoma is still a malignancy that lacks effective therapies. The current study on the function of NK cells in renal cell carcinoma could bring us new ideas about their role in renal cell carcinoma immunotherapy. Though existing some obstacles, the novel CAR-NK therapy may be an effective tool in treating renal cell carcinoma in the near future.
